# Identifying and Validating Tankyrase Binders and Substrates: A Candidate Approach

**DOI:** 10.1007/978-1-4939-6993-7_28

**Published:** 2017-02-09

**Authors:** Katie Pollock, Michael Ranes, Ian Collins, Sebastian Guettler

**Affiliations:** 3 Division of Structural Biology, The Institute of Cancer Research, London, SW7 3RP UK; 4 Division of Cancer Biology, The Institute of Cancer Research, London, SW7 3RP UK; 5 Division of Cancer Therapeutics, Cancer Research UK Cancer Therapeutics Unit, The Institute of Cancer Research, London, SW7 3RP UK

**Keywords:** Tankyrase, PARP, Poly(ADP-ribosyl)ation, Tankyrase-binding peptide motif, Enzyme–substrate relationships, Protein-protein interactions, Protein expression, Protein purification, Fluorescence polarization (FP), Structural biology

## Abstract

The poly(ADP-ribose)polymerase (PARP) enzyme tankyrase (TNKS/ARTD5, TNKS2/ARTD6) uses its ankyrin repeat clusters (ARCs) to recognize degenerate peptide motifs in a wide range of proteins, thereby recruiting such proteins and their complexes for scaffolding and/or poly(ADP-ribosyl)ation. Here, we provide guidance for predicting putative tankyrase-binding motifs, based on the previously delineated peptide sequence rules and existing structural information. We present a general method for the expression and purification of tankyrase ARCs from *Escherichia coli* and outline a fluorescence polarization assay to quantitatively assess direct ARC–TBM peptide interactions. We provide a basic protocol for evaluating binding and poly(ADP-ribosyl)ation of full-length candidate interacting proteins by full-length tankyrase in mammalian cells.

## Introduction

**Fig. 1:**
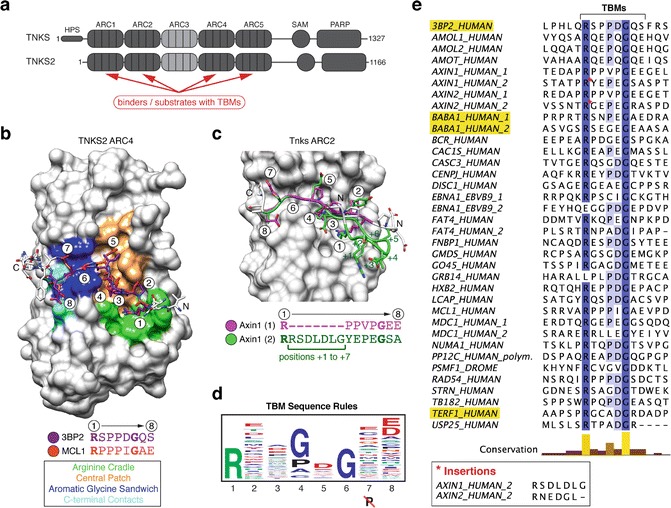
Substrate binding by Tankyrase. (**a**) Domain organization of human tankyrase and tankyrase 2 (modified from [31]). (**b**) and (**c**) Examples for ARC–TBM interactions studied by X-ray crystallography. (**b**) Human TNKS2 ARC4 is shown in surface representation with bound TBM peptidesTankyrase Binders from 3BP23BP2 (SH3BP2, SH3 domain-binding protein 2) and MCL1 shown in stick representation with the core TBM octapeptide colored *purple* and *orange*, respectively, and by heteroatom. TBM amino acid positions (1–8) and sequences shown. The figure was generated by superimposing the ARCs of the ARC4-3BP2 and ARC4-MCL1 crystal structures (PDB accession codes 3TWR and 3TWU, respectively) onto each other and showing ARC4 of the former [7]. The *colored* surface areas represent different contact areas, as indicated, that mediate binding of the TBM peptides (Modified from [7] with permission from Elsevier/Cell Press). (**c**) ARC2 (from ARC2–3) of murine Tnks bound by the N-terminus of murine Axin1 (PDB accession code 3UTM), which contains two TBMs [11]. Each TBM binds one copy each of ARC2 in a dimeric ARC2-3 assembly. The figure was generated by superimposing the two ARC2-3 copies onto each other; the surface of ARC2 bound by the first TBM is shown. TBMs are shown and labeled as in (**b**). The first TBM, shown in *magenta*, consists of a continuous stretch of eight amino acids. In the second TBM, shown in green, the Arg at position 1 is followed by a seven-amino-acid insertion (positions +1 to +7), as indicated in the sequences shown. The peptide insertion forms a loop. (**d**) TBM sequence rulesSequence consensus/rules represented by a sequence logo. (Reprinted from [7] with permission from Elsevier/Cell Press.) (**e**) Sequence alignment of known example TBMs ([7] and references therein, [11, 22, 23, 37–39]), colored by identity with conservation graph, generated with ClustalX and Jalview [40, 41]. UniProtUniProt database IDs are indicated [24]. The *asterisk* indicates insertion sequences in AXIN1 and AXIN2. The TBMs of 3BP2, TRF1 (TERF1_HUMAN), and MERIT40 (BABA1_HUMAN), studied as model TBMs here, are *highlighted*

Poly(ADP-ribosyl)ation (PARylation) constitutes a striking posttranslational modification (PTM) catalyzed by poly(ADP-ribose)polymerase (PARP)Poly(ADP-ribose) polymerases (PARPs) Tankyrase Binders enzymes, which serially transfer ADP-ribose from NAD^+^ NAD+ onto substrate proteins [1]. The resulting long, strongly negatively charged PAR chains provide attachment sites for proteins endowed with PAR-binding modules or directly affect substrate function [2]. PAR can thus act as a scaffolding component as well as a regulatory PTM. PARylation is most commonly associated with nuclear events such as gene regulation and DNA damage repairDNA damage repair, but PAR is found in both the nucleus and the cytoplasm [1, 3]. The PARP tankyrase, of which there are two human paralogs (TNKS/ARTD5, TNKS2/ARTD6Tankyrase (TNKS/ARTD5, TNKS2/ARTD6), *see* Fig. 1a), contributes to both the nuclear and cytoplasmic pools of PAR [4, 5]. The biological roles of the two Tankyrases are largely redundant [6], pointing to shared molecular mechanisms. The recruitment of tankyraseTankyrase recruitment to differentTankyrase Binders protein complexes associated with specific cellular processes and situated at different subcellular locations defines its diverse functions. All Tankyrase binders characterized to date bear a tankyrase-binding motif (TBM)Tankyrase binding motif (TBM) In-silico prediction of Tankyrase binding motifs (TBMs), which in its simplest form consists of six to eight consecutive amino acids [4, 7, 8]. TBMs are recognized by tankyrase’s Ankyrin repeat clusters (ARCs) [7, 9, 10]. Out of the five ARCs, the central one (ARC3) is devoid of a known peptide-binding function while the other four (ARCs 1, 2, 4, and 5) each feature a highly Conserved peptide-binding pocket with similar specificities [7, 10] (Fig. 1a). Given four peptide-binding ARCs, tankyrase recognizes its binders multivalentlyMultivalent binding.

The TBM has been characterized extensively and a consensus sequence Sequence consensus/rules of R-x-x-[small hydrophobic or G]-[D/E]-G-[no P]-[D/E] identified through positional scanning and evaluation of known TBMs [7] (Fig. 1d and e). Paired with structural information from ARC–peptide complexes [7, 11–13], these “sequence rules”Sequence consensus/rules provide a valuable tool for identifying Tankyrase binders. Fig. 1b–e gives examples for X-ray crystal structures describing TBM-ARC interactions, summarize the experimentally derived TBMTankyrase binding motif (TBM) sequence rules, and list known TBMs. Within the motif, the ArgTankyrase Binders residue at position 1, bound in an extensive “Arg cradle,” is essential, as is Gly at position 6, owing to its unique geometry that is adopted when the residue is sandwiched between two aromatic side chains in the ARCAnkyrin repeat clusters (ARCs) (Fig. 1b, d). At positions 2 and 3, where side chains point away from the ARC, a wide range of amino acids is tolerated. At position 4, a small and/or hydrophobic residue binds a tight hydrophobic sub-pocket. Gly, Pro, Ala, and Cys (in this order of preference) have been found suitable by positional scanning [7], and known TBMs mostly feature Pro and Gly, followed by Ala, Val, Leu, and Cys (Fig. 1e). At position 5, Asp is clearly preferred, with Glu ranked second, but Val, Gln, Tyr, Ile, and Cys were all allowed in positional scanning experiments (Fig. 1b, d) [7]. In line with these observations, Asp and Glu are found at position 5 in the vast majority of known TBMs, followed by Gln, Pro, Ile, and Val (Fig. 1e). Positions 7 and 8 tolerate a wide range of amino acids, with the exception of Pro at position 7, where Pro causes an unfavorable distortion of the peptide backbone (Fig. 1d). At position 8, Asp and Glu are most favored, owing to an electrostatic contact with the ARC, and can balance out the negative contribution made by suboptimal residues at other positions (Fig. 1b, d) [7]. Recent structural and biophysical studies suggest that the spatial organization of the five ARCs and the positioning of multiple TBMs within a subset of tankyrase binders encode another determinant of tankyrase binding [13].

Among the tankyrase–substrateTankyrase-substrate relationships most extensively studied to date are those with AXIN (axis inhibition protein, AXIN1Axis inhibition protein 1 (AXIN1), AXIN2), scaffolding proteins involved in Wnt signalingWnt signalling [11, 14], the telomeric protein TRF1TRF1 (TERF1, Telomere repeat binding factor) (telomeric repeat-binding factor 1, official gene name TERF1) [12, 15], and the signaling adaptor protein 3BP23BP2 (SH3BP2, SH3 domain-binding protein 2) (SH3 domain-binding protein 2, official gene name SH3BP2) [7, 16]. Many more Tankyrase binders have been identified, and the list of known interactors keeps expanding [5]. An *in silico* predictionTankyrase Binders of tankyrase bindersIn-silico prediction of Tankyrase binding motifs (TBMs) gives reason to anticipate a broad involvement of Tankyrase in a wide range of biological functions [7]. To understand the complex biological roles of tankyrase, also in light of the considerable interest in tankyrase as a potential therapeutic target [5, 17], we require insights into the complement of tankyrase-binding proteins in the proteome.

Here, we outline a hierarchical three-step candidate approach for identifying Tankyrase binders and substrates, providing further experimental detail on the method reported previously [7]. **Step 1** constitutes TBM prediction, **step 2** the evaluation of TBMs as direct ARC binders by fluorescence polarization (FP), and **step 3** the validation of tankyrase binding and tankyrase-dependent PARylationPoly(ADP-ribosyl)ation (PARylation) in the full-length protein context. We chose two model proteins: the first identified Tankyrase binder, TRF1TRF1 (TERF1, Telomere repeat binding factor) [15], and a novel tankyrase binder, MERIT40MERIT40 (BABAM1, BRISC and BRCA1-A complex member 1) (Mediator of RAP80 interactions and targetingProtein targeting subunit of 40 kDa, official gene name BABAM1), which was identified by the approach presented here [7]. The TBM from 3BP23BP2 (SH3BP2, SH3 domain-binding protein 2) serves as an additional example in the FP assay [7]. As part of **step 2**, we present a general method for the expression and purification of TNKS and TNKS2 ARCs from Escherichia coli (Table 1). ARCs 1, 4, and 5 can be produced as individual domainsTankyrase Binders. ARCs 2 and 3 are insoluble when produced independently; however, they can be produced as a double ARC2-3 construct. Moreover, the entire tankyrase N-termini with all five ARCs can be generated [7, 13]. Proteins are expressed with a cleavableCleavable tag N-terminal His_6_-GST tag His6-GST tag, which enables simple affinity purification, minimally followed by size exclusion chromatography upon tag removal. The subsequent FPFluorescence polarization (FP) assay uses a candidate TBM peptide, synthesized with a fluorescent label such as fluorescein, to directly measure the binding affinity to a tankyrase ARC or a set of ARCs. In this assay, the fluorescent peptide probe is excited by polarized light. The light emitted by an unbound probe loses most of its polarization due to its rapid motion in solution. When bound to an ARC, movement of the peptide is slowed down and a high degree of polarization retained in the emitted light. Titration of tankyrase ARCs at a constant probe concentration allows the dissociation constant (K_d_)dissociation constant (Kd) to be determined [7, 18–20]. Upon confirmation of the isolated TBM, further validation of the candidate Tankyrase binder requires an assessment of tankyrase binding and substrate PARylationPoly(ADP-ribosyl)ation (PARylation) using full-length proteins (**step 3**). We present details for a straightforward assay basedTankyrase Binders on co-expression in HEK293T cellsHuman embryonic kidney (HEK) 293T cells, co-immunoprecipitation and the detection of PAR in the immunoprecipitates, using PAR-binding antibodies PAR-binding antibody [7].

**Table 1:** Human tankyraseankyrase (TNKS/TNKS2) ARC constructs for biophysical assays. The proteins include a non-native, vector-derived GAMGS sequenceTankyrase Binders at the N-terminus that is retained upon cleavage of the affinity tag [7]

ARC construct	Construct boundaries	Molecular weight (kDa)
TNKS ARC1–5	178–958	85.0
TNKS ARC1	178–336	17.2
TNKS ARC2–3	331–645	34.7
TNKS ARC4	646–807	18.0
TNKS ARC5	799–958	17.5
TNKS2 ARC1–5	20–800	85.3
TNKS2 ARC1	20–178	17.7
TNKS2 ARC2–3	173–487	35.3
TNKS2 ARC4	488–649	17.9
TNKS2 ARC5	641–800	17.3

## Materials

Unless the supplier is explicitly mentioned, chemicals are typically obtained from Sigma-Aldrich.

### Protein ExpressionExpression

1. Inducible bacterial expression constructs for affinity-taggedAffinity tag tankyrase ARCs (TNKS: NM_003747.2; TNKS2: NM_025235.2; *see* Table 1 for construct details; *see* **Note** **1**).
2. BL21-CodonPlus (DE3)-RIL E. coli chemically competent cellsBL21-CodonPlus (DE3)-RIL E. coli chemically competent cells (Agilent Technologies) (*see* **Note** **2**).
3. “Lysogeny Broth” (LB)Lysogeny broth (LB) medium agar platesLB agar plates, supplemented with kanamycin (50 μg/mL) and chloramphenicol (34 μg/mL).
4. LB medium (100 mL for overnight starter culture).
5. “Terrific Broth” (TB) mediumTerrific broth (TB) medium (4–8 L for large-scale expression).
6. 1000× stock solutionsTankyrase Binders of antibiotics: 50 mg/mL kanamycin (in H_2_O) and 34 mg/mL chloramphenicol (in isopropanol).
7. Shaking incubators capable of a temperature range of at least 18–37 °C.
8. Erlenmeyer flasks (250 mL) for pre-cultures, baffled Erlenmeyer flasks (2 L) for large-scale expression.
9. Isopropyl β-d-1-thiogalactopyranoside (IPTG9. Isopropyl β-D-1-thiogalactopyranoside (IPTG), 1 M stock solution)
10. Refrigerated centrifuge for harvesting large volumes of bacterial cultures (4000 × *g*, e.g., Beckman Coulter Avanti J-26XP with JLA 8.1000 rotor).
11. Liquid nitrogen bath.
12. 50 mL Falcon tubes or plastic film with thermal sealer for storage of bacterial pelletsExpression.

### Protein purification

1. Protease inhibitors Protease inhibitors, such as Pierce protease inhibitor tablets, EDTA (Thermo Fisher Scientific).
2. Lysozyme, 40 mg/mL stock.
3. Sonicator fitted with a large probe or homogenizer capable of breaking bacterial cellsProtein purification.
4. Ultra-filtered H_2_O.
5. Cell lysis buffer: 50 mM Tris–HCl pH 7.5, 500 mM NaCl, 5 mM β-mercaptoethanolmercaptoethanol. Add protease inhibitor protease inhibitor tabletsTankyrase Binders and lysozyme (100 μg/mL final concentration) immediately before use.
6. Refrigerated centrifuge for removing insoluble lysate fraction (30,000 × *g*, e.g., Beckman Coulter Allegra 64R with F0650 rotor).
7. 5.0 μm syringe filter units.
8. 5 mL HisTrapHisTrap nickel affinity column HP Ni^2+^ affinity column (GE Healthcare, *see* **Note** **3**).
9. Peristaltic pumpProtein purification.
10. Vacuum pump and bottle filters (0.22 μm) for filtering and degassing buffers.
11. Buffer A for Ni^2+^ affinity column: 50 mM Tris–HCl pH 7.5, 500 mM NaCl, 5 mM β-mercaptoethanol β-mercaptoethanol, 10 mM imidazole pH 7.5—filtered and degassed.
12. Buffer B for Ni^2+^ affinity column: 50 mM Tris pH 7.5, 500 mM NaCl, 5 mM β-mercaptoethanol, 250 mM imidazole pH 7.5—filtered and degassed.
13. FPLCFast protein liquid chromatography (FPLC) system with buffer gradient capabilitiesTankyrase Binders, UV absorbance detector and fraction collector (e.g., ÄKTA Purifier, GE Healthcare).
14. 5 mL HiTrap Q HP column (GE Healthcare).
15. Buffer A for Q column: 50 mM Tris–HCl pH 7.5, 100 mM NaCl, 5 mM β-mercaptoethanol—filteredmercaptoethanol and degassed.
16. Buffer B for Q column: 50 mM Tris–HCl pH 7.5, 1.5 M NaCl, 5 mM β-mercaptoethanol—filtered and degassed.
17. Dialysis buffer: 50 mM Tris–HCl pH 7.5, 100 mM NaCl, 5 mM β-mercaptoethanol (see Note 11)Protein purification.
18. Dialysis tubing, 3500 Da molecular weight cutoff (MWCO).
19. Dialysis tubing clips.
20. 2 L beaker, magnetic stirrer plate, stirrer bar.
21. TEV proteaseTobacco etch virus (TEV) protease, 5 mg/mL stock.
22. 15 mL spin protein concentrator, 3000 Da MWCO for single-ARC constructs, 10,000 Da MWCO for double-ARC constructs, 30,000 Da MWCO for ARC1-5 constructsProtein purification.
23. Refrigerated centrifuge for concentrating protein (3200 × *g*, e.g., Beckmann Coulter Allegra X12-R centrifuge with SX4750 swinging bucket rotor).
24. Refrigerated centrifugeTankyrase Binders for removing precipitate prior to size exclusion chromatography (18,000 × *g*, e.g., Eppendorf 5417R with F45-30-11 rotor).
25. HiLoad 16/600 Superdex 75 or 200 pg size exclusion column (GE Healthcare, *see* **Note** **4**).
26. Tris(2-carboxyethyl)phosphine (TCEP), 0.5 M stock (*see* **Note** **5**).
27. Size exclusion buffer: 25 mM HEPES-NaOH pH 7.5, 100 mM NaCl, 2 mM TCEP—filtered and degassed (*see* **Notes** **6** and **11**)Protein purification.
28. 96-deep-well blocks for fraction collection, or fraction collector tubes (depending on the format of the fraction collector).
29. UV spectrophotometer.
30. 4× SDS sample buffer.
31. 15% polyacrylamide gels for SDS-PAGE.
32. Protein standard for SDS-PAGE.
33. Coomassie stain for SDS-PAGE gels.
34. 15 mL and 50 mL Falcon tubes Protein purification.
35. Thin-walled individual 0.2 mL PCR tubes for flash-freezingProtein flash freezing protein aliquots.
36. Liquid nitrogen bath.

### Fluorescence Polarization (FP) AssayFuorescence

1. Plate reader capable of taking FP measurements (e.g., BMG Labtech POLARstar Omega).
2. Appropriate wavelength filters for chosen Fluorophore, one corresponding to excitation wavelength, and two (ideally a matched pair with identical optical properties), corresponding to the emission wavelength. Here, we use a 485 nm excitation filter and two matched 520 nm emission filtersTankyrase Binders for fluorescein.
3. Opaque, black, 384-well, non-binding, flat-bottom plates, either in standard format (e.g., 781,900, Greiner Bio-One) or in small-volume format (e.g., 784,900, Greiner Bio-One). The latter are particularly useful if limited protein is available.
4. Microplate centrifuge (1000 × *g*, e.g., Beckman Coulter Allegra X-12R with SX4750 swinging bucket rotor, fitted with microplate inserts)
5. FP assay buffer: 25 mM HEPES–NaOH pH 7.5, 100 mM NaCl, 1 mM TCEPTris(2-carboxyethyl)phosphine (TCEP), 0.05% w/v 3-[(3-Cholamidopropyl)dimethylammonio]-1-propanesulfonate hydrate (CHAPS) (*see* **Notes** **7** **and** **11**).
6. Fluorescently tagged peptide corresponding to TBM of potential Tankyrase binder, 2× stock (50 nM for a final assay concentration of 25 nM) in FP assay buffer. In the present examples (3BP23BP2 (SH3BP2, SH3 domain-binding protein 2), MERIT40MERIT40 (BABAM1, BRISC and BRCA1-A complex member 1), TRF1TRF1 (TERF1, Telomere repeat binding factor)), fluorescein and 5(6)-carboxyfluorescein are used as fluorophores. The peptidesTankyrase Binders have been described previously [7]. We recommend peptides of minimally the TBM octapeptide with one flanking amino acid on either side. The peptides used here are longer (*see* Fig. 3b for peptide sequences). The fluorescein fluorophore is linked via β-Ala, which also provides an additional linker to minimize potential steric interference of the fluorophore with the peptide-ARC interaction (*see* **Note** **8**).
7. Tankyrase ARC protein: 2× stock of twofold dilution series, 0–400 μM, (final assay concentrations of 0–200 μM protein) in FP assay bufferFuorescence.
8. Software for curve fitting and analysis curve fitting and analysis (e.g., GraphPad Prism 6).

### Binding and PARylationPoly(ADP-ribosyl)ation (PARylation) of Full-Length Candidate Proteins by Tankyrase in Cells

1. Mammalian expression constructsMammalian expression constructs for epitope-tagged (e.g., MYC_2_) tankyrases (TNKS: NM_003747.2; TNKS2: NM_025235.2) and epitope-tagged (e.g., FLAGFLAG tag) tankyrase-binding protein candidatesTankyrase Binders (here for TRF1TRF1 (TERF1, Telomere repeat binding factor)/TERF1: NM_017489.2 and MERIT40/BABAM1: NM_001033549.2), corresponding empty vectors as controlsEmpty vector control.
2. QuikChange Lightning mutagenesis kit (Agilent Technologies) or individual components from other sources for site-directed mutagenesis.
3. Mutagenesis primers to mutate putative TBM (recommended mutation: G6R).
4. PCR thermal cycler, standard setup and reagents for recombinant DNA techniquesPoly(ADP-ribosyl)ation (PARylation) Tankyrase.
5. Human Embryonic Kidney (HEK) 293T cells (ATCC).
6. 10 cm and 15 cm cell culture dishes.
7. Hemocytometer or automated cell counter.
8. Dulbecco’s Modified Eagle’s Medium (DMEM).
9. Fetal bovine serum (FBS).
10. HumidifiedTankyrase Binders cell culture incubators at 37 °C, 5% CO_2_ CO2.
11. 0.2% Versene in PBS (137 mM NaCl, 2.7 mM KClPotassium chloride (KCl), 8 mM Na_2_HPO_4_, 1.5 mM KH_2_PO_4_ KH2PO4, 537 μM EDTA, 80 μM phenol redPhenol, final pH adjusted to 7.2, sterilization-autoclaved; all reagents cell-culture grade).
12. 0.05% trypsin in Versene (137 mM NaCl, 2.7 mM KCl, 8 mM Na_2_HPO_4_, 5.5 mM D-glucose d-glucose, 1.5 mM KH_2_PO_4_ KH2PO4, 25 mM Tris, 1% phenol red, 0.5 g trypsin (1:250) per 1 L, 137 μM streptomycin sulfate, 168 μM benzyl penicillin, final pH adjusted to 7.5, filter-sterilized using a 0.22 μm filter).
13. Ultra-filtered sterile H_2_OPoly(ADP-ribosyl)ation (PARylation) Tankyrase.
14. Calcium phosphate transfection Calcium phosphate transfection reagents (all cell culture grade):
  - 2× HEPES-Buffered Saline (HBS): 50 mM HEPES2-[4-(2-hydroxyethyl)piperazin-1-yl]ethanesulfonic acid (HEPES) buffer, 10 mM KCl, 280 mM NaCl, 1.5 mM Na_2_HPO_4_, 12 mM glucose, final pH adjusted to 7.05 with KOHPotassium hydroxide (KOH), filter-sterilizedFilter sterilisation and storedTankyrase Binders at 4 °C.
  - 25 mM chloroquine diphosphate, filter-sterilized and stored at −20 °C (1000× stock).
  - 2 M CaCl_2_ Calcium chloride (CaCl2), filter-sterilizedFilter sterilisation and stored at 4 °C.
15. Phosphate-Buffered Saline (PBS).
16. Cell scraperPoly(ADP-ribosyl)ation (PARylation) Tankyrase.
17. Refrigerated centrifuge for collecting mammalian cells (300 × *g*, e.g., Beckmann Coulter Allegra X-12R with SX4750 swinging bucket rotor).
18. Radioimmunoprecipitation assay (RIPA) buffer: 50 mM HEPES–NaOH pH 7.5, 150 mM NaCl, 1% Triton X-100, 0.5% sodium deoxycholate, 0.1% SDSSodium Dodecyl Sulfate (SDS), 1 mM DTT, 2 μM ADP-HPD PARG inhibitor PARG inhibition Adenosine 5′-diphosphate(hydroxymethyl)pyrrolidinediol (ADP-HPD, PARG inhibitor) and protease inhibitors (e.g., Pierce protease inhibitor tablets, EDTA, Thermo Fisher Scientific). Add DTT, ADP-HPD, and protease inhibitors immediately before use.
19. Sonicator fitted with small probe.
20. Refrigerated centrifuge for clearing lysates and settling affinity resin (800–18,000 × *g*, e.g., Eppendorf 5417R with F45-30-11 rotor).
21. Anti-FLAG M2 Agarose resinAnti-FLAG M2 Agarose resin.
22. Vacuum pump with inlet trapTankyrase Binders and collection flask for aspirating buffer.
23. SDS-PAGE gels (e.g., 4–15% Tris–glycine polyacrylamide gradient gels for excellent resolution).
24. Electrophoresis apparatus for the above and appropriate power supply.
25. 4× SDS sample buffer.
26. Protein standard for SDS-PAGEPoly(ADP-ribosyl)ation (PARylation) Tankyrase.
27. Nitrocellulose transfer membrane.
28. Wet transfer Western blotting apparatus and appropriate power supply.
29. Ponceau S solution.
30. Nonfat dry milk powder.
31. Mouse monoclonal anti-FLAGMouse monoclonal anti-FLAG antibody, HRP conjugate M2 HRP-conjugated antibody (here 1 mg/mL), mouse monoclonalMouse monoclonal anti-MYC antibody, HRP-conjugate anti-MYCAnti-MYC antibody (9E10) HRP-conjugated antibody (here 1 mg/mL), rabbit polyclonalRabbit polyclonal anti-PAR antibody anti-PAR (4336-BPC-100, Trevigen, concentration not specified by supplier), goat anti-rabbit IgG (H+L) secondary antibody, HRP-conjugate (here 0.8 mg/mL) (*see* **Note** **9**).
32. ECLEnhanced chemiluminscence (ECL) Western blotting substratePoly(ADP-ribosyl)ation (PARylation) Tankyrase.
33. X-ray film for Western blot detection or alternative ECL detection setup.

## Methods

The details of many standard experimentalTankyrase Binders methods not addressed here can be found in [21].

### Predicting Tankyrase binders

Experimental approaches such as yeast-two-hybrid analysis [22] or co-immunoprecipitation coupled with tandem mass spectrometry [23] have been used to identify proteins present in complexes with tankyrase. It is desirable to pinpoint those proteins that directly interact with tankyrase. The existence of putative TBMs is the most reliable known indicator for direct interaction. Based on previously identified TBM sequence rulesSequence consensus/rules and structural information, we provide a guide to identifying strong TBM candidates for further validationValidation of candidate TBM. A previously generated list of ranked octapeptide motifsPeptide motif across the entire human proteome can be used [7]. Here, we recapitulate a simple step-by-step approach for identifying candidate TBMs without the need for specialized bioinformatics beyond the use of established programs and databases.

1. Screen your protein of interest for potential TBMs. In an initial step, a search for Arg and Gly spaced 6 amino acids apart (i.e., with four amino acids in between, RxxxxG) will suffice. *See* **step 4** below for atypical TBMs.
2. Using UniProtUniProt database (http://www.uniprot.org), assess the topology of the proteinProtein topology and whether the candidate motif would be located in the cytoplasm or nucleoplasm, accessible to tankyrase [24]. The relevant information can be found under *Subcellular location > Topology*.
3. Assess the occurrence of disordered stretches in your protein of interest. TBMs must be present in unstructured regionsProtein unstructured region of the protein to be accessible to Tankyrase and must not be part of a stable α-helix or β-strand. Secondary structure information, obtained from the Protein Data Bank [25], is available in UniProt (http://www.uniprot.org) under *Structure > Secondary structure* [24]. It remains possible that TBMs are present within loops of structured protein domains. If no structural informationTankyrase binders is available, a disorder predictor such as IUPred (http://iupred.enzim.hu) may be used to obtain an indication of protein disorder [26].
4. Assess the short-listed sequences for their suitability as TBMs. REAGDGEE has been found to be the optimal 8-amino-acid TBM by positional scanning [7], but many TBMs only span six amino acids (positions 1–6). While the sequence rulesSequence consensus/rules from positional scanning prove valuable in predicting TBMs, it should be noted that some of their aspects may be specific to the context of the 3BP2 TBM peptide3BP2 (SH3BP2, SH3 domain-binding protein 2), which positional scanning was performed on [7]. Whereas Arg and Gly at positions 1 and 6, respectively, are essential, other residues are important as well, with the exception of positions 2 and 3, which can likely be ignored since they do not contribute to binding and mainly fulfil spacing roles (Fig. 1b, d). Please refer to the motif description under Subheading 1 for the assessment. Of note, a large or strongly charged amino acid at position 4 very likely disqualifies the TBM candidate as a genuine Tankyrase binder. Furthermore, Asp and Glu at position 8 can compensate for unfavorable amino acids at other positions [7], as seen in one of the TBMs of AXIN1/2, where a suboptimal Val at position 4 appears to be counterbalanced by a Glu at position 8 (Fig. 1e) [7, 11]. Interestingly, a second TBM in AXIN1/2 shows that the Arg, normally at position 1, can be placed further N-terminal, resulting in looping-out of the “inserted” residues (Fig. 1c, e). Likewise, the E3 ubiquitin ligase RNF146 has been proposed to contain numerous atypical TBMs with a nonconventional positioning of the Arg [27], and it is possible that a distal Arg is required for weak binding of the TBM of GRB14 to Tankyrase (Fig. 1e) [7, 8, 28]. Thus, the possibility of a distal Arg residue should be taken into consideration. It is likely that such extended TBMs display a weaker affinity for tankyrase. Numerous Tankyrase binders contain multiple TBMs, such as AXIN1/2 [11] and MERIT40 [7] (*see* below). Since avidity effects likely contribute to binding of these proteins to tankyrase, the affinity of some of these individualTankyrase binders TBMs for the ARC may be lower, and this may be reflected in deviations from the “optimal” sequence.

### Validating TBMsValidation of candidate TBMFuorescence by Fluorescence Polarization (FP)

#### Expression of Tankyrase ARCs

cDNA sequences encoding TNKS or TNKS2 ARC constructs are cloned into a pETM-30-2 expression vector (or similar vector) to generate fusion proteins with an N-terminal His_6_-GST tag His6-GST tag followed by a TEV proteaseTobacco etch virus (TEV) protease cleavage site (ENLYFQG) [7]. *See* Table 1 for suggested ARC boundaries [7]. Chemically competent BL21-CodonPlus (DE3)-RIL E. coli cellsBL21-CodonPlus (DE3)-RIL E. coli chemically competent cells for protein expression are transformed with sequence-validated plasmids. A single colony is selected to inoculate an overnight pre-culture, which is then used to inoculate the larger expression cultures (typically 4–8 L). The cultures are grown shakingTankyrase Binders at 37 °C to exponential phase, then cooled at 4 °C for 30 min before induction with IPTG overnight at 18 °C. The cells are then collected by centrifugationTBMs Validation of candidate TBM Fuorescence. Purification can either follow immediately, or cell pellets can be stored at −80 °C until processed for purificationExpression ARCs.

1. Transform chemically competent BL21-CodonPlus (DE3)-RIL E. coli cellsBL21-CodonPlus (DE3)-RIL E. coli chemically competent cells with Tankyrase ARC expression constructs by heat shock. Plate cells on LB agar plates containing suitable antibiotics, here kanamycin (50 μg/mL) and chloramphenicol (34 μg/mL) (*see* **Notes** **1** and **2**). Incubate overnight at 37 °C.
2. Pick a single colony from the plate and inoculate an overnight pre-culture of LB medium (100 mL) supplemented with 50 μg/mL kanamycin and 34 μg/mL chloramphenicol. Incubate overnight in a shaking incubator at 37 °CTBMs Validation of candidate TBM Fuorescence.
3. Add 5 mL of the pre-cultureExpression ARCs to each liter of TB mediumTerrific broth (TB) medium supplemented with 50 μg/mL kanamycin and 34 μg/mL chloramphenicol. Generally, the yield for individual TNKSTankyrase Binders and TNKS2 ARC constructs is 6–10 mg/L of culture.
4. Incubate cultures at 37 °C with shaking at 180 rpm until an optical density at 600 nm (OD_600_) of approximately 2.0 is reached. Remove cultures from incubatorTBMs Validation of candidate TBM Fuorescence and cool them at 4 °C for 30 min (*see* **Note** **10**).
5. Add IPTG to a final concentration of 0.5 mM (0.5 mL of 1 M stock per liter of culture) to induce protein production. Incubate cultures at 18 °C with shaking overnight (≈15 h).
6. Collect cells by centrifugation in a precooled centrifuge at 4 °C for 30 min at 4000 × *g*.
7. Discard supernatant. The cells can be lysed for immediate purification. Alternatively, pellets can be flash-frozen in a liquid nitrogen bath for storage at −80 °C until requiredExpression ARCs.

#### Purification of Tankyrase ARCs

**Fig. 2:**
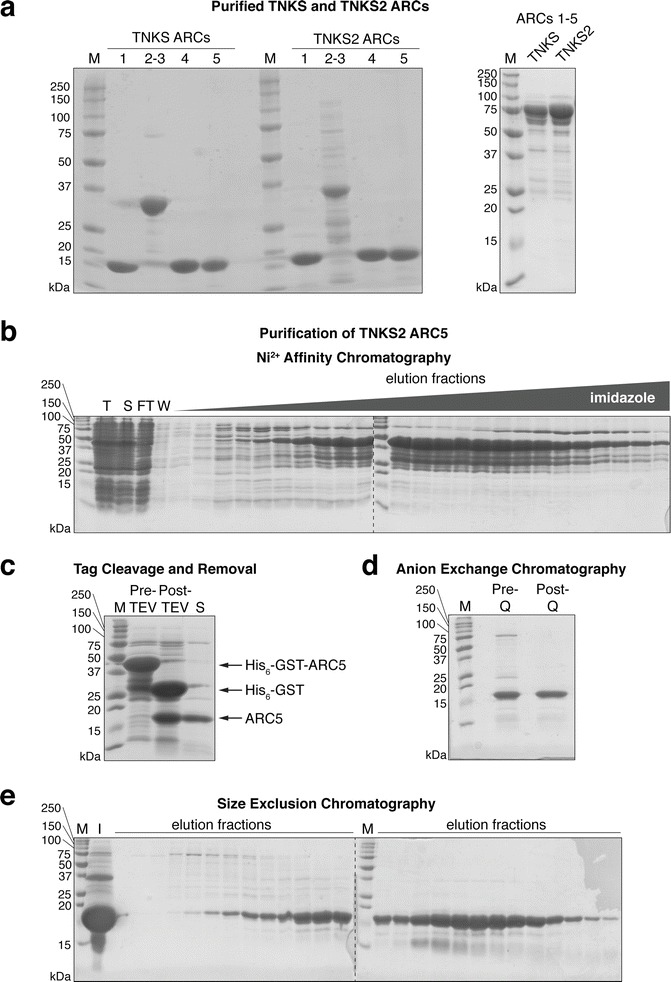
Purification of tankyrase ARCs. (**a**) 5 μg purified TNKSTankyrase Binders and TNKS2 ARCs were resolved on 15% Tris–glycine polyacrylamide gels for SDS-PAGE and the gels stained with Coomassie. M, marker. *See* Table 1 for construct boundaries. (b)-(e) Step-by-step purification of a representative ARC, TNKS2 ARC5. (**b**) Ni^2+^ affinity purification of His_6_-GST-TNKS2 ARC5 fusion protein. T, total lysate; S, soluble lysate; FT, flow-through; W, wash. (**c**) Removal of the His_6_-GSTGST tag His6-GST tag affinity tag by TEV cleavage and a second Ni^2+^ affinity purification step. Pre-TEV, protein before TEV cleavage; Post-TEV, protein after TEV cleavage; S, pooled protein after subtraction of affinity tag. (**d**) Anion exchange chromatographyAnion exchange chromatography (Mono Q column). Pre-Q, protein before anion exchange chromatography; Post-Q, protein after anion exchange chromatography. (**e**) Size exclusion chromatography. M, marker; I, input

A similar procedure is followed for the purification of all tankyrase ARCs. All buffers are ice-cold, and workTBMs Validation of candidate TBM Fuorescence is performed at 4 °C or on ice throughout. In the first step, the protein is affinity-purified by immobilized metal affinity chromatography (IMAC)immobilized metal affinity chromatography (IMAC) using a Ni^2+^ affinity column. The affinity tag is then cleaved overnight using TEV proteaseTobacco etch virus (TEV) protease, under simultaneousTankyrase Binders dialysis to remove imidazole from the elution step. A second Ni^2+^ affinity chromatography step separates the cleaved tag from the protein. An optional anion exchange chromatographyAnion exchange chromatography (Mono Q column) step removes further impurities. Finally, size exclusion chromatography yields high-quality purified protein for downstream applications. The general procedure is applicable to all TNKS and TNKS2 ARCs with small changes (Fig. 2a; *see* **Note** **11**).

1. Dissolve protease inhibitor protease inhibitor tablets in the required volumeTBMs Validation of candidate TBM Fuorescence ARCs of lysis buffer (approximately 20 mL of buffer for every 10 g of cell pellet) and add lysozyme (100 μg/mL final) in a large glass beaker containing a magnetic stirrer bar. Add frozen cell pellet and stir gently until cells are completely thawed and a homogeneous suspension is obtained.
2. Break cells by using a large-tip sonicator at 40% amplitude output, using a 5 s on, 5 s off pulse, for a total of 5 min “on” time. Keep cells on ice continuously to avoidARCs overheating (*see* **Note** **12**). Take a sample of the total lysate for subsequent analysis by SDS-PAGE (9 μL lysate + 3 μL 4× SDSSodium Dodecyl Sulfate (SDS) sample buffer).
3. Remove cell debris and insoluble material by centrifugation at 30,000 × *g* for 30 min. If lysate is still cloudyTankyrase Binders, centrifuge for a further 30 min until clear.
4. While the lysate is being cleared, prepare a 5 mL HisTrapHisTrap nickel affinity column Ni^2+^ affinity column by washing with 10 column volumes (CV) of H_2_O and then equilibrating with 10 CV of buffer A for Ni^2+^ affinity column, using a low-pressure peristaltic pump, set to a flow rate of 2 mL/min (*see* **Note** **3**).
5. Decant the supernatant into a beaker, and sonicate on ice, 5 s on, 5 s off for a total of 20 s “on” time to further break up genomic DNAGenomic DNA (gDNA) Genomics and avoid clogging the filter in the next stepTBMs Validation of candidate TBM Fuorescence ARCs.
6. Filter the supernatant through 5.0 μm syringe filter units. Take a sample of the soluble lysate for subsequent analysis by SDS-PAGE (9 μL lysate + 3 μL 4× SDS sample buffer). Load the cleared lysate on the equilibrated HisTrap columnHisTrap nickel affinity column using the peristaltic pump as before (*see* **Note** **13**). The His_6_-GST-taggedGST tag protein will bind to the immobilized Ni^2+^ ions. Wash the column with 20 CV of buffer A (100 mL) to remove unbound contaminants. Collect lysate flow-through and the wash buffer in case the protein was not bound or was released during the wash, and take samples for analysis by SDS-PAGE (9 μL flow-through or wash + 3 μL 4× SDS sample buffer).
7. Attach the HisTrap columnHisTrap nickel affinity column to an FPLCFast protein liquid chromatography (FPLC) system, and elute the His_6_-GST-ARC proteinTankyrase Binders with the following three-step protocol, at a flow rate of 2 mL/min, collecting 1.5 mL fractions in a 96-deep-well block:
  step 1: 100% buffer A for 2 CVARCs.
  step 2: gradient from 0–100% buffer B over 20 CV.
  step 3: 100% buffer B for 4 CV.
8. Take samples of each peak elution fraction (9 μL of eluate + 3 μL 4× SDS sample buffer). Analyze the samples taken in **steps 2** and **6** (2 μL of sample for lysates and flow-through, 5 μL of sample for wash) and the peak elution fractions (5 μL of sample) by SDS-PAGE (Fig. 2b).
9. Pool fractions containing ARC TBMs Validation of candidate TBM Fuorescence fusion protein. Take a sample of the pooled fractions (9 μL of protein + 3 μL 4× SDS sample buffer). Add TEV protease (approximately 100 μL of 5 mg/mL stock for every 25 mL fusion protein solution) to cleave the tag.
10. Soak Dialysis tubing (3500 Da MWCO) in dialysis buffer, and seal one end with a dialysis membrane clip. Transfer the protein into the dialysis tubing and seal the other end. More than one dialysis tube may be required. Dialyze overnight in a 2 L glass beaker containing dialysis buffer, with gentle stirring at 4 °C. Avoid contact between the stirrer bar and the dialysis tubing to prevent ruptureTBMs Validation of candidate TBM Fuorescence.
11. Pour content of dialysis tubing into 50 mL Falcon tubes. Centrifuge at 3200 × *g* for 15 min to remove any precipitate that may have formed. Take a sample of the protein post-TEV cleavage (9 μL of protein + 3 μL 4× SDS sample buffer). White precipitate often forms overnight with the change to low-salt buffer. SDS-PAGE analysis of the precipitate reveals it to be enriched in a contaminant (not shown).
12. Meanwhile, wash a 5 mL HisTrap columnHisTrap nickel affinity column with 10 CV of H_2_OTankyrase Binders. Next, equilibrate the column in 10 CV of dialysis buffer. FilterARCs the supernatant through a 0.22 μm syringe filter and load it onto the equilibrated HisTrap columnHisTrap nickel affinity column using the low-pressure peristaltic pump. Collect the flow-through. The untagged ARC TBMs Validation of candidate TBM Fuorescence protein will pass through, while the cleaved tag (or residual His_6_-GST-ARC fusion protein) will be retained on the column (*see* **Note** **14**). Wash with 2 CV of dialysis buffer to fully remove all ARC protein from the column, collecting the wash. Take samples of the flow-through and wash (9 μL of protein + 3 μL 4× SDS sample buffer).
13. Analyze 5 μL of the pre-TEVTobacco etch virus (TEV) protease sample, post-TEV sample and flow-through/wash samples from the second Ni^2+^ column by SDS-PAGE to assess successful tag removal and separation (Fig. 2c).
14. Take the protein-containing samples (flow-through/wash) and concentrate the protein to about 20 mL by centrifuging in a 3000 Da MWCO spin concentrator at 3200 × *g* for 30 min at a time in a swinging bucket rotor. Mix by gently pipetting up and downTBMs Validation of candidate TBM Fuorescence to prevent a steep concentrationTankyrase Binders gradient from forming in the concentrator. Take a sample of the concentrated protein (9 μL of protein + 3 μL 4× SDS sample buffer).
15. The following anion exchange chromatographyAnion exchange chromatography (Mono Q column) step is optional, but was found to increase the purity of ARCARCs proteins, especially TNKS2 ARC5, by removing a higher-molecular-weight impurity. Using the peristaltic pump at 2 mL/minARC TBMs Validation of candidate TBM Fuorescence, wash a 5 mL HiTrap Q HP column with 5 CV of H_2_O, followed by 5 CV of buffer B for Q column, and then equilibrate with 10 CV of buffer A for Q column. Pass the concentrated protein over the column, and collect the flow-through. Wash the column with 2 CV of buffer A for Q column, also collecting the flow-through. ARCs do not bind to the Q column; however, the impurity does and will be retained on the column (*see* **Note** **15**). Take a sample of the combined flow-through and wash (9 μL of protein + 3 μL 4× SDS sample buffer). Analyze 5 μL of the samples from **step 14** and this step by SDS-PAGE as above (Fig. 2d).
16. Concentrate the protein as in **step 14**, to ≤2 mL.
17. Centrifuge protein in a microcentrifugeTankyrase Binders tube at 18,000 × *g* for 10 min to remove any particles before size exclusion chromatography.
18. Load the concentrated protein onto an equilibrated size exclusion column connected to an FPLC systemTBMs Validation of candidate TBM Fuorescence, using a 2 mL sample loop (*see* **Note** **4**). Elute the protein isocratically, using 1.2 CV of size exclusion buffer. Collect 1 mL fractions in a 96-deep-well block.
19. Take samples of the input material and peak fractions (9 μL input or fraction + 3 μL 4× SDS sample buffer). Analyze 5 μL of the samples by SDS-PAGE (Fig. 2e).
20. Combine fractions containing pure protein. Wash the membrane of a spin concentrator by spinning through one concentrator volume of H_2_O to remove any stabilizing agents such as glycerol and azide. Now concentrate the protein using the pre-washed spin concentrator as described above (**step 14**) until a concentration of >0.5 mM is achievedARCs (solubility of multi- ARC constructs can be lower). The concentration can be determined by measuring the UV absorbance at 280 nm and using the molar extinction coefficient calculated by ProtParam (*http://web.expasy.org/protparam/*) [29].
21. Aliquot the protein into small volumes in thin-walled PCR tubes and flash-freeze them in a liquid nitrogen bath. Frozen protein aliquots can be stored at −80 °C until future use.

#### Fluorescence PolarizationFluorescenceTBMValidation of candidate TBM Assay for TBM Validation

This assay measures the direct binding of a fluorescently labeled TBM candidate peptide to a Tankyrase ARC [7]. The ARC protein is titrated at a constant peptideTankyrase Binders probe concentration, and the change in polarization (ΔFP) is measured and plotted. Plotting ΔFP values against [ARC] enables a dissociation constant (K_d_)dissociation constant (Kd) to be calculated. In the present example, we use fluorescein peptide labels, but the general method can be adapted for different fluorophores.

1. Ensure the plate reader is set up for fluorescence polarization, with the correct filters for the fluorophore TBMs Validation of candidate TBM Fuorescence. The fluorescein example uses a 485 nm excitation filter and two 520 nm emission filters, one parallel and one perpendicular to the plane of the linearly polarized light used for excitation.
2. Dissolve the lyophilized peptide probe in FP assay buffer, to approximately 100 μM. The concentration can be measured spectrophotometrically (*see* **Note** **16**). Here, we measure the absorbance of the peptide stock at 492 nm and use a molar extinction coefficient for fluorescein of ε_492_ = 83,000/M/cm [30]. The concentrated probe can then be aliquoted into small volumes (5–10 μL) and flash-frozen in liquid nitrogen for long-term storage at −80 °C, to be thawed when required.
3. Determine the optimal probe concentration to use. This will depend on the sensitivity of the plate reader. FPFluorescence TBM Validation of candidate TBM values should be independent of the probe concentration used, and give a robust signal windowTankyrase Binders for an unbound vs. protein-bound peptide. Make 2× stocks of a twofold dilution series of probe in FP buffer, from 0–800 nM (10 concentrations + no peptide). It is often easier to make a 1/100 dilution of the concentrated peptide stock to avoid pipetting small volumes. Total sample volumes per well are either 15 or 50 μL (*see* **step 5** below).
4. Prepare a 2× protein stock solution, by diluting tankyrase ARC protein in FP buffer to 20 μM (10 μM final protein concentration). The protein concentration should be in vast excess over the peptide probe to obtain a first impression of the anticipated signal window between free and protein-bound peptide probeTBMs Validation of candidate TBM Fuorescence.
5. Plate out the assay samples in technical duplicate (*see* Table 2, **Note** **17**). Small-volume plates will require 15 μL per well (7.5 μL of each stock), standard-volume plates 50 μL per well (25 μL of each stock). Mix the probe stock solution 1:1 with either FP buffer for the peptide probe-only titration or protein stock for the titration in the presence of proteinFluorescence TBM Validation of candidate TBM. Add a “blank” well with FP buffer only. A well with 5 nM fluorescein (literature FP of 35 millipolarization units, mP) can be used as a standard to automatically set the gain adjustmentTankyrase Binders of the plate reader. Temporarily seal the plate with adhesive film or a lid and spin at 1000 × *g* for 1 min in a swinging bucket rotor with plate inserts. Incubate in the dark at room temperature for 30 min to ensure binding equilibrium is reached.

**Table 2:** Example plate layout for establishing the probe concentration in a fluorescence polarizationFluorescence polarization (FP) assay.Rows B and C correspond to 3BP2 peptide titrations (here up to 400 nM) in the absence of protein, while rows D and E correspond to 3BP2 peptide titrations in the presence of protein. Numbers correspond to final fluorophore concentration (nM). Blank wells contain buffer alone. Standard wells contain 5 nM of free fluorescein. Wells along the edge of the microplate (row A and column 1) are left empty to minimize the “edge effect” (*see* **Note** **17**)

	1	2	3	4	5	6	7	8	9	10	11	12	13	14
A														
B		0	0.78	1.56	3.13	6.25	12.5	25	50	100	200	400	Blank	Standard
C		0	0.78	1.56	3.13	6.25	12.5	25	50	100	200	400	Blank	Standard
D		0	0.78	1.56	3.13	6.25	12.5	25	50	100	200	400	Blank	Standard
E		0	0.78	1.56	3.13	6.25	12.5	25	50	100	200	400	Blank	Standard
6. Set the gain adjustment of the instrument. The POLARstar Omega automatically calculates the gain by scaling the FP value to 35 mP for the wells containing 5 nM free fluorescein. 50 flashes per well is a good starting point to minimize fluorophore bleaching, and means each data point will be the average of 50 measurements.
7. Read the plate and calculate FP in mP. Some plate readers and software packages will automatically blank-correct and calculate the FP values. If not, subtract the averaged F_parallel and F_perpendicular readings obtained for the blank wells from the corresponding individual values of the other wells. FP values are calculated using the following equation:
$$ FP \ (mP)=\frac{\left( F\_ parallel- F\_ perpendicular\right)}{\left( F\_ parallel+ F\_ perpendicular\right).} $$
polarized light *used for excitation. mP, millipolarization units.*
8. Export the FP data and raw fluorescence intensity values. Plot the data (average FP vs. [probe]) using GraphPad Prism or your data analysis program of choice (Fig. 3a).

**Fig. 3:**
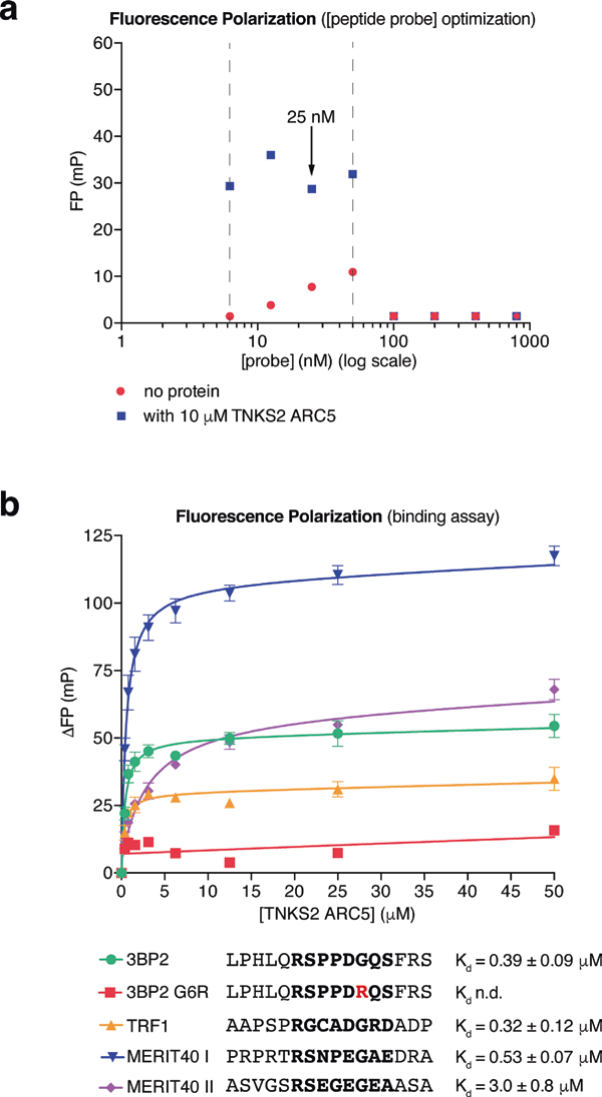
Assessing ARC–TBM peptide interactionsARC-peptide interactions by fluorescence polarizationFluorescence. (**a**) Optimization of the peptide probe (3BP2) concentration for the FP binding assay. Data are from one experiment performed in technical duplicate with mean FP values plotted. The dashed vertical lines indicate the suitable peptide probe concentration range. 25 nM was chosen for the subsequent assays. (**b**) FP binding assays for the TBM peptides from TRF1, MERIT40, and 3BP2 (WT positive control and G6R negative control). Peptide sequences (octapeptide in bold) and affinities for TNKS2 ARC5 are indicated. n = 3 separate experiments; error bars, SEM. The error values for the dissociation constants correspond to the standard error of the fit in nonlinear regression. n.d., not determined (no binding curve)
9. Determine the minimum probe concentrationTankyrase Binders required. Raw fluorescence intensity values of >10× the blank reading are necessary to calculate accurate FP values. In the example shown in Fig. 3a, fluorescence intensity values measured with probe concentrations below 6.25 nM were too low and therefore excluded.
10. Determine the maximum probe concentration. The FP values will drop sharply when the signal overloads the detectors. In this case, both detectors reach saturation, so the fluorescence intensities registered in the parallel and perpendicular channels will be identical, and hence the FP calculated will be zero. In the example shown in Fig. 3a, 50 nM is the maximum usable peptide probe concentration. FP readings between 6.25 and 50 nM probe are approximately constant.
11. Peptide probe concentrations should be kept as low as reasonably possible. Thus, the peptide concentration can be ignored in K_d_ calculations. 25 nM probe was chosen for the protein titration experiments shown belowTBMs Validation of candidate TBM Fuorescence. Prepare a 2× probe stock solution (50 nM in FP buffer). Prepare a twofold dilution series of tankyrase ARC protein from 0–400 μM (0–200 μM final concentration) in FP buffer. The maximum protein concentrationTankyrase Binders may be loweredFluorescence TBM Validation of candidate TBM if B_*max*_ is reached at low protein concentrations, i.e., in the case of high affinity. An initial maximum [ARC] of 200 μM ensures that binding can be detected for weak binders as well.
12. Set up a binding experiment for K_d_ determination. Mix the probe and protein stock solution 1:1 to give a final well volume of 15 μL (7.5 μL of each stock solution, small-volume plates). Centrifuge the plate as in **step 5** above. Incubate the plate in the dark at room temperature for 30 min to achieve equilibrium before measurement.
13. Calculate FP as in **step 7** above and export the raw fluorescence and calculated FP values.
14. Enter the individual FP values into GraphPad Prism or your data analysis program of choice.
15. Baseline by subtracting the FP value for the zero-protein well from all other values in that row, to obtain ΔFP values. Calculate the mean of the technical duplicates. Plot ΔFP against [protein]. Perform a nonlinear regression analysis, using a one-site total binding model:
*ΔFP = B*_max_ × *[ARC]/(K*_d_ *+ [ARC]) + NS* × *[ARC] + Background*
*B*_*max*_ *denotes the maximum* Tankyrase Binders specific binding *in mP, where the binding curve saturates; K*_d_ dissociation constant slope nonspecific binding baseline *ΔFP value, which should be zero. Although it is possible to constrain the background to zero, it is advisable to retain the background term since the baseline is defined by more values than that from the zero-protein sample.*
  Non-binding peptides will give a straight line of Nonspecific binding. Binding peptides will give a logarithmic curve (Fig. 3b). This analysis will yield a K_d_ value.
16. Repeat the experiment to obtain pooled data from (typically three) separate experimentsFluorescence TBM Validation of candidate TBM. In each separate experiment, technical duplicates are combined into a single data point. Error bars (SEM) are calculated for the mean data points from the separate experiments (Fig. 3b). The peptides from 3BP23BP2 (SH3BP2, SH3 domain-binding protein 2), TRF1TRF1 (TERF1, Telomere repeat binding factor), and MERIT40 (two peptides) bind TNKS2 ARC5 with the indicated affinities, while no binding is detectable for the G6R negative control peptide from 3BP2 (Fig. 3b).

### Validation of TankyrasePoly(ADP-ribosyl)ation (PARylation) Binding and PARylation in Full-Length Protein Context

This final section outlines a step-by-step approach to assess and validate the full-length candidate protein interaction with full-length tankyrase and the extent of its tankyrase-dependent PARylationTankyrase Binders. Mammalian expression constructsMammalian expression constructs for MYC_2_-taggedMYC tag TNKS2 (pLP-dMYC SD-TNKS2)Tankyrase Poly(ADP-ribosyl)ation (PARylation) and FLAG- or FLAG_3_-taggedFLAG tag candidate interacting proteins (pCMV-FLAG-MERIT40 and pLP-tripleFLAG SD-TRF1) are generated. Standard site-directed mutagenesis is performed to mutate the Gly at position 6 of the FP-validated TBMs Validation of candidate TBM Fuorescence polarization (FP) to Arg. The FLAG-taggedFLAG tag candidate proteins and corresponding TBM mutant derivatives are next used as “baits” in co-immunoprecipitation experiments, to assess their binding to and their PARylation by MYC_2_-TNKS2, using catalytically inactive TNKS2 (G1032W) as control [31].

#### Co-expression of TNKS2 and MERIT40 or TRF1 in HEK293T CellsHuman embryonic kidney (HEK) 293T cells

1. Grow a sufficient number (here two) 15 cm cell culture platesCulture plates (96-well, 24-well, 6-well, white, black) of HEK293T cells to a cell density of ≈90% confluence in standard DMEM media supplemented with 10% FBS.
2. Remove the media and gently rinseTankyrase Binders the cell culture plates with 10 mL of Versene to remove residual media, which could impede the efficient dissociation of the cells by trypsin.
3. Add 3 mL of trypsin–Versene to each plate and place the plates back in the incubator for 2 min. GentlyTankyrase Poly(ADP-ribosyl)ation (PARylation) tap the side of the plates to help the cells detach from the plate and dissociate from each other.
4. Add 10 mL of DMEM media with 10% FBS to each plate; the FBS will inactivate the trypsin. Pipette the cell suspension up and down a few times to ensure proper dissociation of cell clumps into a homogenous cell suspension.
5. Measure the cell densityTankyrase Poly(ADP-ribosyl)ation (PARylation) Co-expression MERIT40 TRF1 Human embryonic kidney (HEK) 293T cells, using either a hemocytometer or automated cell counter. Add 6 × 10^6^ cells each to the required number of 10 cm cell culture platesCulture plates (96-well, 24-well, 6-well, white, black) for the co-transfection of different DNA constructs (Table 3). Add 10 mL of DMEM media with 10% FBS to each plate and incubate cells overnight.

**Table 3:** Chart for co-transfection of HEK293T cellsFluorescence polarization (FP) for co-immunoprecipitation. The indicated FLAG/FLAG_3_-taggedFLAG tag constructs (5 μg) are used as baitsTankyrase Binders in co-immunoprecipitation with the indicated MYC_2_-taggedMYC tag TNKS2 constructs (5 μg). The G-to-R mutation at position 6 of the TBM efficiently abolishes the ARC–TBM peptide interactionARC-peptide interactions [7]. A G1032W mutation abolishes both poly- and mono(ADP-ribosyl)ation by TNKS2 [31]. Each "+" sign denotes a single co-transfection setup

	empty FLAG vector	MERIT40 WT	MERIT40 G33R	MERIT40 G53R	MERIT40 GG33/53RR	TRF1 WT	TRF1 G18R
empty MYC_2_ vector	−	+	−	−	−	+	−
TNKS2 WT	++	+	+	+	+	+	+
TNKS2 G1032W	−	+	−	−	−	+	−
6. The next day, co-transfectTankyrase Binders cells with 5 μg of each DNA construct (thus 10 μg total) per plate (Table 3). Pipette 5 μg of each DNA constructCo-expression MERIT40 TRF1 Human embryonic kidney (HEK) 293T cells in a 15 mL Falcon tube, add 250 μL of 2 M CaCl_2_ CaCl2 followed by 1.75 mL H_2_O and mix thoroughly. Subsequently, add 2 mL of 2× HBS, mix the solution thoroughly by pipetting up and down and incubate for 5–10 min at room temperature (*see* **Note** **18**).
7. Replace the growth media from the 10 cm plate with 15 mL of fresh growth media supplemented with 25 μM chloroquine. Chloroquine inhibits degradation of endocytosed DNA and can increase transfection efficiency [21].
8. Gently add the transfection mixTankyrase Poly(ADP-ribosyl)ation (PARylation) (4 mL each, dropwise) to each plate and incubate transfected cells for 24 h.
9. The next day, remove the media and add 10 mL of ice-cold PBS. Harvest the cells by gently scraping them off the plate (*see* **Note** **19**). Collect the scraped cell suspension in a 15 mL Falcon tube and pellet the cells by centrifugation at 300 × *g* for 5 min at 4 °C. Discard the supernatant; snap-freeze cell pelletCo-expression MERIT40 TRF1 Human embryonic kidney (HEK) 293T cells in liquid nitrogen and store at −80 °C until further use.

#### Co-immunoprecipitation

1. Add 1 mL of ice-cold RIPA bufferRadioimmunoprecipitation assay (RIPA) buffer (with freshly added DTT and protease inhibitors protease inhibitors) to each cell pellet and resuspend cellsTankyrase Binders on ice. The freeze–thaw and the detergents will break open the cells. Transfer the lysates to 1.5 mL microcentrifuge tubes and incubate on ice for 5 min for extraction.
2. Sonicate lysates for 6 s on ice, using a small tip sonicator at 25% amplitude output to shear chromatin.
3. Clear cell lysates by high-speed centrifugation at 18,000 × *g* for 15 min at 4 °C and transfer supernatants to new 1.5 mL microcentrifuge tubes.
4. Transfer 30 μL of each cleared lysate to a new microcentrifuge tube (**input samples**) and add 10 μL 4× SDS sample buffer. Boil samples at 95 °C for 5 min, collect by brief centrifugationTankyrase Poly(ADP-ribosyl)ation (PARylation) Co-immunoprecipitation, and store at −20 °C until analysis, if required.
5. Pre-equilibrate a sufficient amount of anti-FLAG M2 Agarose resinanti-FLAG M2 Agarose resin (25 μL of packed-volume resin per immunoprecipitation sample plus a minimum of 10% dead volume) with RIPA buffer and incubate resin with cleared lysates for 2 h on a rotating wheel at 4 °C.
6. Gently settle the resin by centrifugation at 800 × *g* for 5 min at 4 °C and remove supernatant using a vacuum pump.
7. Wash the resin by adding 1 mL of RIPA buffer to each sample and incubating microcentrifugeTankyrase Binders tubes for 5 min on a rotating wheel at 4 °C.
8. Settle the resin as before (**step 6**) and repeat the wash step four more times.
9. After the last wash step, gently remove as much of buffer as possible and add 25 μL of 2× SDS sample buffer. Boil samples at 95 °C for 5 min, collect briefly by centrifugationTankyrase Poly(ADP-ribosyl)ation (PARylation) Co-immunoprecipitation and store at −20 °C until analysis, if required (**immunoprecipitate (IP) samples**).

#### SDS-PAGE and Immunoblotting

**Fig. 4:**
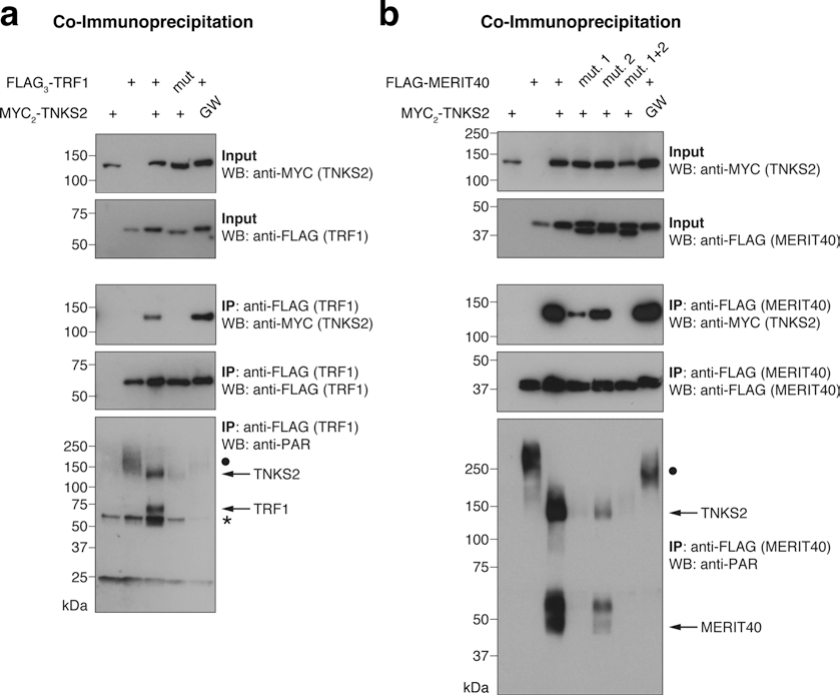
Assessing Tankyrase binding and PARylation by tankyrase in the full-length protein context. (**a**) FLAG_3_-TRF1, either in its wild-typeTankyrase Binders form or as a G18R TBM mutant (“mut.”), was co-expressed with the indicated MYC_2_-TNKS2 constructs, either in wild-type form or as a G1032W PARP-inactive mutant (“GW”) [31]. FLAG-TRF1 was immunoprecipitated and input and immunoprecipitate (IP) samples analyzed by SDS-PAGE and Western blotting as indicated. “•” in the anti-PAR blot labels a high-molecular-weight PARylated species that appears to be antagonized by MYC_2_-TNKS2 overexpression. “*” in the anti-PAR blot denotes a nonspecific band. (**b**) Same analysis as in (**a**) with FLAG-MERIT40, either in wild-type form or as a G33R (“mut. 1”), G53R (“mut. 2”), or GG33/53RR (“mut. 1 + 2”) TBM mutant. MERIT40 appears as a doublet, most likely reflecting differentially phosphorylated species [32]. *See* **Note** **22** on attributing PAR signals to candidate proteins

1. Resolve 10 μL of the boiled input (**step 4** above) and IP (**step 9** above) samples on a pre-cast 4–15% Tris–glycine polyacrylamide gradient gel for SDS-PAGE analysis.
2. Transfer proteins from the gel onto a nitrocellulose membrane using a wet-transfer blotting systemTankyrase Poly(ADP-ribosyl)ation (PARylation). Ponceau S can be used to assess the transfer quality.
3. Incubate the nitrocellulose membrane for at least 1 h at room temperature on a horizontal shaking platform in 5% dry milk powder in PBS to block the membraneTankyrase Binders to reduce nonspecific binding of antibodies during the subsequent immunodetection steps.
4. Incubate the membranes with the required antibody at the appropriate dilution in 5% milk/PBS overnight at 4 °C on a horizontal shaking platform. (Antibody dilutions: anti-FLAG HRP-conjugated antibody, 1:1000; anti-MYCAnti-MYC antibody HRPHorseradish peroxydase (HRP)-conjugated antibody, 1:1000; anti-PAR, 1:1000)SDS-PAGE Immunoblotting Tankyrase Poly(ADP-ribosyl)ation (PARylation).
5. Wash the membrane three times for 5 min with copious amounts PBS + 0.1% Tween 20 on a horizontal shaking platform.
6. As required, incubate the membrane with the matching HRP-coupled secondary antibody, here diluted 1:5000 in 5% milk/PBS for 1 h at room temperature on a horizontal shaking platform (*see* **Note** **20**).
7. Repeat wash **step 5**.
8. Develop Western blot by incubating membrane with ECL Western blotting substrate following the manufacturer’s instructions.
9. Detect chemiluminescence signal by exposing membrane to X-ray film in a dark room and developTankyrase Binders the film with an X-ray film developer (*see* **Note** **21**). Fig. 4 shows the TBM FLAG_3_-TRF1 and FLAG-MERIT40 with MYC_2_-TNKS2, and the TNKS2-dependent PARylationTankyrase Poly(ADP-ribosyl)ation (PARylation) of both TNKS2 interactors, in addition to TNKS2 auto-PARylation (see Note 22). Both MERIT40 TBMs contributeSDS-PAGE Immunoblotting to Tankyrase binding (Fig. 4b).

## Notes

1. We recommend using vectors with a kanamycin selection marker. Ampicillin hydrolysis by secreted β-lactamase and under low pH increases the proportion of cells lacking the plasmid, which decreases protein yield [33].
2. RIL cells contain additional tRNAs for codons of Arg, Ile, and Leu that are otherwise rare in E. coli. The plasmid bearing these genes contains a chloramphenicol selection marker.
3. We recommend using one 5 mL column per 4 L expression culture. If using larger volumes, connect additional column(s) in series. Ni^2+^ affinity columns can easily be reconstituted by stripping from and re-charging with Ni^2+^ as per the manufacturer’s instructions.
4. Use a Superdex 200 column for ARC1-5 constructs. All other ARC constructs are sufficiently small for the Superdex 75 column.
5. TCEPTris(2-carboxyethyl)phosphine (TCEP) is more stable than DTT and not volatile, unlike β-mercaptoethanol β-mercaptoethanol or DTT. However, TCEP is acidic and will affect the final pH of the buffer unless the 0.5 mM stock solution is pH-adjusted with NaOH. To keep costs down, TCEPTankyrase Binders is only used in the final purification step.
6. HEPES2-[4-(2-hydroxyethyl)piperazin-1-yl]ethanesulfonic acid (HEPES) buffer is preferred for the final protein and in experiments due to its lower temperature dependency compared with Tris [34]. Tris was chosen for the affinity purification step due to its weak interaction with Ni^2+^, which would help decrease background (contaminant) binding [35]. If HEPES buffer is used in the affinity purification step, the imidazole concentration may need to be increased to achieve comparably low background binding.
7. Detergent is used to reduce nonspecific binding and surface tension that may interfere with fluorescence intensity readings in the plate format. The choice of detergent and its concentration is empirical. We have also had good experience with using 100 μg/mL bovine serum albumin (BSA), but use CHAPS3-[(3-Cholamidopropyl)dimethylammonio]-1-propanesulfonate hydrate (CHAPS) here since it is more effective at reducing nonspecific binding.
8. Keeping the fluorescently labeled peptides as short as possible increases the ΔFP signal window between the bound and unbound states. Ideally, peptides should be HPLC; however, this is not always realistically achievable, especially if large numbers of peptides are to be compared and no access to in-house solid-state peptide synthesis is available. To save costs, peptidesTankyrase Binders can be used at non-HPLC-purified grade, in which case a capping step is strongly recommended after each amino acid coupling reaction to prevent peptide synthesis intermediates from being linked to the fluorophore in the final coupling step and affecting the assay [7].
9. Alternatively, fluorescently labeled antibodiesAntibody can be used for detection with appropriate fluorescence imaging systems. Instead of the anti-PAR antibody, an anti-pan-ADP-ribose or anti-poly(ADP-ribose) binding reagents (MABE1016 and MABE1032, respectively, Millipore) may be explored.
10. Compared to LB, TB is richer and enables higher cell densities in the log phase of growth. Cooling the cultures before IPTG induction slows down expression, thereby facilitating correct protein folding and increasing protein solubility.
11. For ARC2-3 and ARC1-5 constructs described here, a minimal NaCl concentration of 300 mM needs to be maintained, compared to 100 mM for all single-ARC constructs, and glycerol may further help stabilize the protein [7].
12. For large volumes, it may be easier to lyse cells in two batches. Alternative disruption techniques can be used, such as homogenization by an Avestin EmulsiFlex homogenizer.
13. If available with the FPLC setup, a superloop or, preferably, a sample pump can be used to load the column.
14. Depending on the ARC construct and the final concentration of imidazole in the dialysis buffer, ARCs can bind weakly to the Ni^2+^ column even after tag cleavageTankyrase Binders. They can be eluted with a further imidazole gradient. While this adds one more step to the purification protocol, it enables even higher purities to be achieved.
15. The Q column step can also be performed before concentration, directly using the flow-through from the second Ni^2+^ affinity column; however, prior concentration saves time in loading the column.
16. Confirm the pH of the peptide stock solution before measuring the concentration. Acidity, for example due to residual trifluoroacetic acidTrifluoroacetic acid (TFA) from the peptide synthesis, will strongly affect fluorophore absorption.
17. We recommend leaving wells in the outermost rows and columns empty to reduce the microplate “edge effect,” a discrepancy in readings between the central and peripheral wells [36].
18. It is important to add the different transfection reagents in the specified order to ensure proper calcium phosphate–DNA particle formation.
19. Avoid using micropipette tips to transfer the cell suspension after scraping. The small opening of the tips can cause cells to break due to shearing. You can cut off the tips to avoid this risk.
20. For directly HRP-coupled antibodies, azide as a preservative should be avoided as it inhibits HRPHorseradish peroxydase (HRP) activityTankyrase Binders.
21. The Western blot protocol can be adapted for film-free chemiluminescence detection or fluorescence detection.
22. Attributing a PAR signal to a particular protein by molecular weight may be challenging, in part due to possible PAR-induced mobility shifts in SDS-PAGE. Since the tankyrase substrate candidates are immunoprecipitated from cell lysates, it is possible that the observed PAR signal corresponds to other PARylated proteins in a protein complex. Ultimate confirmation of substrates can be obtained from experiments with purified proteins or PAR site mapping by mass spectrometry, for example.
